# Effects of artificial management on culm properties of *Dendrocalamus brandisii*

**DOI:** 10.3389/fpls.2024.1397686

**Published:** 2024-06-18

**Authors:** Yingdan Yan, Chuanfei Yang, Yufang Wu, Jiaxin Liu, Shuguang Wang

**Affiliations:** ^1^ Key Laboratory for Sympodial Bamboo Research, Faculty of Life Sciences, Southwest Forestry University, Kunming, China; ^2^ Science and Technology Innovation Team of National Forestry and Grassland Administration, Southwest Forestry University, Kunming, China; ^3^ Biological Research and Utilization Innovation Team in Bamboo Resources of Yunnan Province, Southwest Forestry University, Kunming, China

**Keywords:** *Dendrocalamus brandisii*, artificial management, fiber morphology, chemical components, carbohydrates storage

## Abstract

The artificial cultivation and management were extensively carried out in *Dendrocalamus brandisii* stands. However, the influences of artificial management on the anatomical and chemical characteristics of the bamboo culms were unknown. In this study, the fiber morphology, chemical composition and sugar accumulation of the *D. brandisii* culms with management and without management were compared in order to determine the influences of artificial management on bamboo culms. The results indicated that artificial management had a significant influence on the fiber morphology, resulting in shorter fiber length, larger L/T ratio, and smaller W/Lu value. However, the management not only increased the contents of moisture, ash, SiO_2_, and extractive, but also increased the holocellulose contents and decreased the lignin contents, as compared to those without management. Moreover, the management significantly increased the endogenous carbohydrates storage in the culms so as to improve the shoot production. The bamboos under management conditions could still be utilized as a raw material for papermaking. This provided a theoretical basis for the artificial management of *D. brandisii* stands.

## Introduction

As a traditional raw material, bamboo has a long history of application in various industries in China, such as traditional handicrafts, paper making and construction (Jahan et al., 2015; Chen et al., 2019; Maulana et al., 2020; Wang et al., 2022). With the wide application of bamboo, more and more works are focused on the anatomical, physical and chemical properties of bamboo culms. The forestry resources in China are limited, but the bamboo resources are abundant (Fei and Qi, 2020). Bamboo has the advantage of rapid growth and high yield, and therefore, the management of bamboo forests can significantly reduce deforestation (Fei et al., 2016).

*Dendrocalamus brandisii*, a large sympodial bamboo species of Poaceae, Bambusoideae, is a tropical and subtropical suitable bamboo species, and is widely distributed in southern Yunnan. The bamboo shoots are bursting with freshness, offering a sweet and delectable taste. Not only are they highly nutritious, but they also boast certain health care functions. The sturdy texture and sound mechanical properties of the culms render it an excellent choice for manufacturing various products such as bamboo flooring, furniture, daily necessities, building materials, and agricultural goods. Compared with *D. latiflorus*, *Phyllostachys edulis*, *Ph. violascens* and other bamboo species, few basic studies were done on *D. brandisii*, which were mainly concentrated on the bacterial community of bamboo of forest land (Zhu et al., 2023), cutting and rapid propagation (Zhang and Wang, 2004; Yang, 2023), introduction and cultivation (Zheng, 2016; Chen, 2021), nutrient composition and utilization of bamboo shoots (Chen et al., 2018; Ji et al., 2018; Pei et al., 2018), nutrient composition and dietary fiber of bamboo leaves (Ma et al., 2021; Li et al., 2023a; Wang et al., 2024) and silicon fertilizer application and phytolith formation (Zhan et al., 2018, 2019). However, systematic research is lacking on the anatomical characteristics, sugar storage and metabolism, physicochemical properties and timber use as well as papermaking ability of *D. brandisii* culms under artificial management.

During the field management works in the *D. brandisii* forests in Pu ‘er, local bamboo farmers typically perform thinning and selective cutting of bamboo culms every winter. Consequently, only 3–4 culms of 1- and 2-year-old bamboos were retained as maternal bamboos in each cluster, while all culms of 3-years-old and older are removed. Meanwhile, the top of the retained culms were trimmed, ensuring that their height did not exceed 2.0 meters. This was done to promote the sprouting of lateral branch buds and to expand the photosynthetic areas and then further to increase the shoot production. However, it remains unclear whether these artificial management techniques have significant effects on carbohydrate storage in bamboos, as well as their impact on the anatomical and chemical characteristics of the culms. To investigate the impact of artificial management on *D. brandisii* bamboo culms, the current study collected samples of different ages with and without artificial management. A comprehensive analysis of anatomical, physiological, and chemical indicators was conducted to compare and contrast the effects of these management practices on the bamboos. This comparative analysis not only aids in understanding the impact of artificial management on bamboo culms but also aids in determining their suitability for processing in industrial applications.

## Materials and methods

### Materials

The culms of *D. brandisii* were collected from bamboo garden in Shijiazhai village, Pu ‘er City, Yunnan Province (101°N, 22.79°E) in November 2022. A total of 9 culms with no selective cutting and upper sections trimming from three age classes (1, 2 and 3 years old) of *D. brandisii* were collected as the control groups, in which the internodal lengths ranged from 300 to 500mm and the internodal diameters ranged from 65 to 85mm. Meanwhile, a total of 6 culms with their upper sections trimmed from two age classes (1 and 2 years old) of *D. brandisii* were obtained as the treatment group, with the internode lengths of 200–300mm and diameters of 55–65mm.

Internodes were consecutively numbered from bottom to top for each culm, which was then divided into three portions (3rd for bottom, 8th for middle and 15th for top portions) of every age group and chipped into strips (Wang et al., 2011). Under artificial management conditions, the top sections of the 2-year-old culms were trimmed, and the 3-year-old culms were also selectively cut. Therefore, the data related to these two parts were excluded from the subsequent experiments. For physiological analysis, the internode samples of bottom were stored at -80°C. For fiber morphological determination, the internode samples were cut into strips with the size of 2 cm × 2 mm. For anatomical observations, the internode samples were chipped into strips (2cm × 2cm × 4cm) and fixed in FAA solution (1.85% formaldehyde + 45% alcohol + 0.25% acetic acid) and then were stored in a mixture of 50% alcohol and 50% glycerin (Lybeer et al., 2006).

## Methods

### Anatomical observations

The softened samples were cut into sections with 20 µm thickness by using a sliding microtome (Leica SM2010R, Germany). For the starch grain localization, the method of periodic acid-Schiff (PAS) reaction was employed. The sections were soaked in 0.5% KIO_4_ for 10 minutes, followed by the Schiff reagent for 20 minutes, and were then stained in Fast green FCF (Ameresco 0689, Solarbio, Beijing, China). For analysis on the lignin deposition, the phloroglucinol-HCl staining method was employed to observe the degree of lignification according to the method of Carmen et al. (2002), and the lignified cells were stained red or purplish red.

For measuring fiber dimensions, the internode samples were macerated with Jeffery’s solution (1 ∶ 1 mixture ratio of 10% nitric acid and 10% chromic acid) for 36 to 72 h as suggested by Wilson (1954). A minimum of 100 intact fibers from each sample were measured for fiber length, tangential diameter, wall thickness, and lumen diameter using a microscope (Phoenix PH100–3B41L-IPL, China). The slenderness ratio (L/T = length/tangential diameter) and Runkel ratio [W/Lu = (2 × wall thickness)/lumen diameter] were calculated according to the measured fiber dimensions.

### Chemical properties

For chemical analysis, the strips were oven dried at 105°C for 20 min, and then dried at 60°C to constant weight, and then were ground in a Wiley mill. The ground material was placed in a shaker and particles that passed through no. 40 mesh sieve but retained on no.60 mesh were used for the subsequent chemical analysis. The moisture content (MC) was calculated as the following formula: MC=(fresh weight - dry weight)/fresh weight × 100%. The contents of ash, SiO_2_, water extractives, 1% NaOH extractives, alcohol-benzene extractives, holocellulose, acid-soluble lignin and acid-insoluble lignin were determined according to GB/T 2677.3–93 (1993), GB/T 2677.4–93 (1993), GB/T 2677.5–93 (1993), GB/T 10741–89 (1989), GB/T 2677.10–1995 (1995), GB/T10337–2008 (2008) and GB/T 2677.8–94 (1994).

### Carbohydrates determination

In bamboo internodes, the stored carbohydrates mainly include soluble sugar and starch, and the contents of which were determined according to the method of Glassop et al. (2007) and Dubois et al. (1956). About 0.5 g samples were ground to powder in a mortar and pestle using liquid nitrogen, and then were extracted with deionized water. The supernatants collected by centrifugation at 4000 rpm for 10 min were reacted with 5% phenol and 98% sulfuric acid for half an hour to determine the soluble sugar content. The absorbance at 485 nm was determined by spectrophotometer (UV-8000, MWTASH, shanghai, China). An additional 9.2 mol/L perchloric acid was added during the reaction to determine the starch content.

The nonstructural carbohydrate (NSC) values were calculated as the sum of soluble sugar and starch contents. Each determination was repeated three times.

### Data analysis

All data presented were derived from the average of three independent biological replicates. The data was processed and mapped by Excel 2010 software. The data from the experiments were statistically analyzed and compared by one-way ANOVA using the least significant difference method (LSD) to determine the level of significance at P ≤ 0.05 by using SPSS 22.0 software (SPSS, Inc., Chicago, IL, USA). The results were plotted using GraphPad Prism (Prism 8.0.2, San Diego, USA). All the data were presented as means ± standard deviation.

## Results

### Anatomical changes of *D. brandisii* culms with and without artificial managements

**Figure 1:**
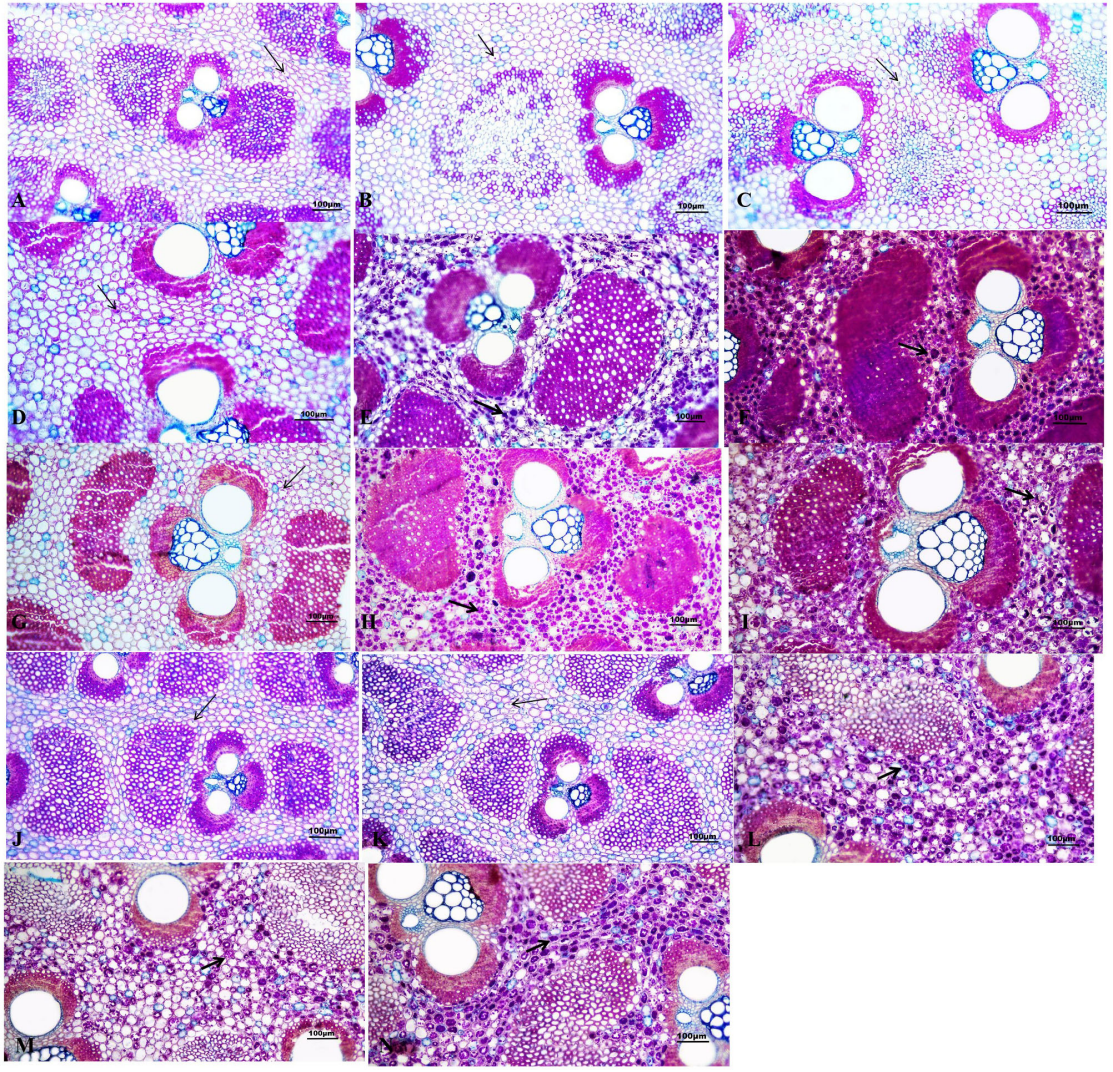
Distribution of starch grains in *D. brandisii* culms without management and with management. Bar = 100 µm. **(A)** Bottom of 1-year-old culms without management. **(B)** Middle of 1-year-old culms without management. **(C)** Top of 1-year-old culms without management. **(D)** Bottom of 2-year-old culms without management. **(E)** Middle of 2-year-old culms without management. **(F)** Top of 2-year-old culms without management. **(G)** Bottom of 3-year-old culms without management. **(H)** Middle of 3-year-old culms without management. **(I)** Top of 3-year-old culms without management. **(J)** Bottom of 1-year-old culms with management. **(K)** Middle of 1-year-old culms with management. **(L)** Top of 1-year-old culms with management. **(M)** Bottom of 2-year-old culms with management. **(N)** Middle of 2-year-old culms with management.

According to the result of the PAS reaction, numerous starch grains were observed and mainly localized in the parenchyma cells between vascular bundles in the culms with and without artificial managements (**Figure 1**). More starch grains were shown in the top than in the middle and bottom in all aged bamboos (**Figures 1A–N**). Meanwhile, the number of starch grains also gradually increased with age in all three internodes. More starch grains were observed in the culms with management compared to those without any management, especially in the 2-year-old bamboos (**Figures 1D–F**).

**Figure 2:**
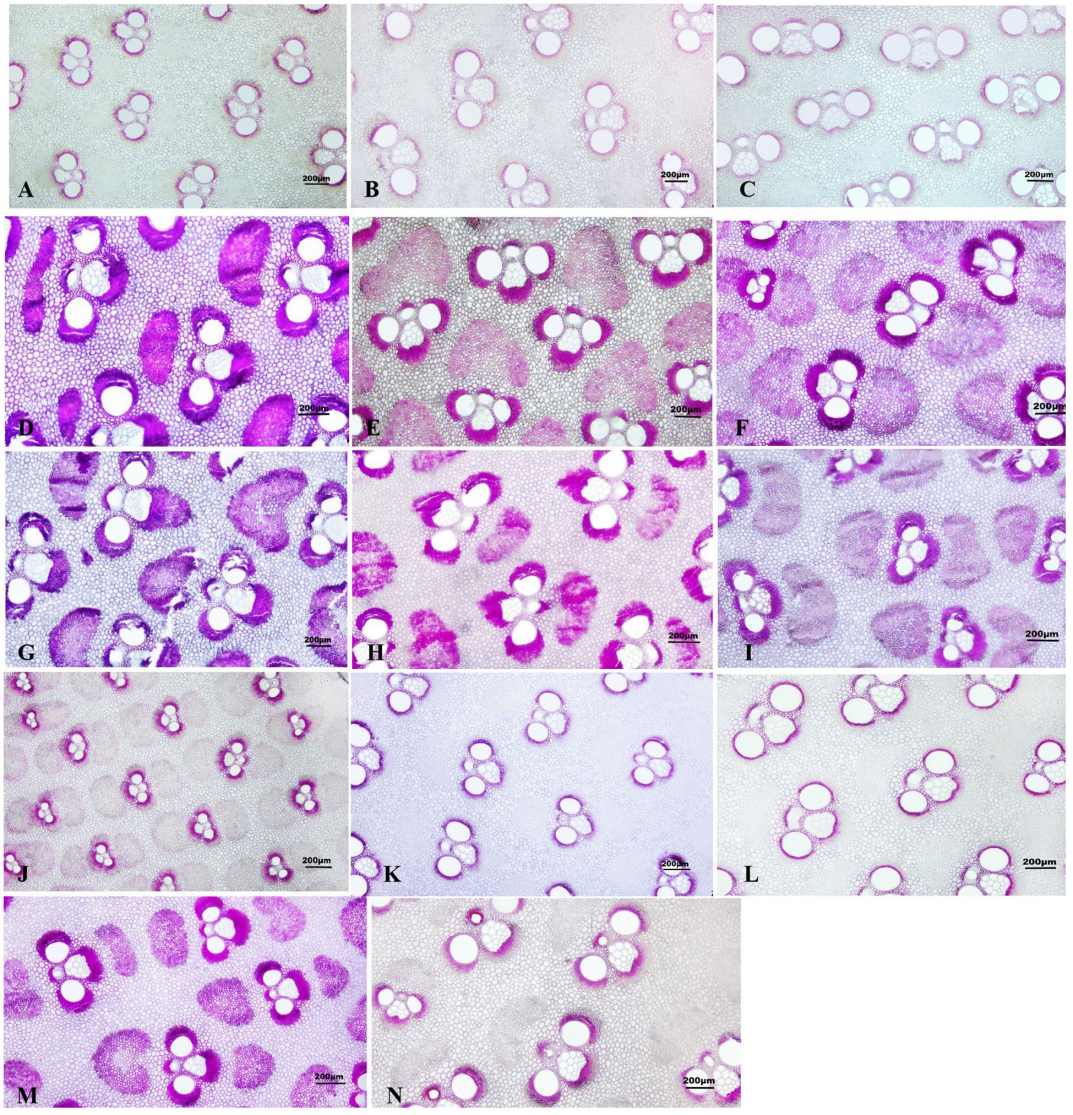
Lignification of *D. brandisii* culms without management and with management. Bar = 200 µm. **(A)** Bottom of 1-year-old culms without management. **(B)** Middle of 1-year-old culms without management. **(C)** Top of 1-year-old culms without management. **(D)** Bottom of 2-year-old culms without management. **(E)** Middle of 2-year-old culms without management. **(F)** Top of 2-year-old culms without management. **(G)** Bottom of 3-year-old culms without management. **(H)** Middle of 3-year-old culms without management. **(I)** Top of 3-year-old culms without management. **(G)** Bottom of 1-year-old culms with management. **(K)** Middle of 1-year-old culms with management. **(L)** Top of 1-year-old culms with management. **(M)** Bottom of 2-year-old culms with management. **(N)** Middle of 2-year-old culms with management.

It was observed that the lignification degree of the bottom was higher than the middle and the top in all aged bamboos, and the lignification degree of culms gradually increased with age (**Figure 2**).The result also indicated that the culms with management had lower lignification degree compared to those without management (**Figures 2A–E, J–N**).

### Changes in fiber morphology of *D. brandisii* culms with and without artificial management

The morphology of fiber has a significant impact on the physical properties of pulp (Zhan et al., 2017a). The fiber morphological characteristics of *D. brandisii* were measured individually and compared between culms of different ages under both artificial management conditions and without any management. These characteristics mainly included fiber length, tangential diameter, wall thickness, lumen diameter, slenderness ratio, and Runkel ratio (**Tables 1**, **2**).

**Table 1:** Changes of the fiber length, tangential diameter and L/T ratio of *D.brandisii* in the two management modes.

Management	Age (year)	Position	Length (µm)			Tangential diameter (µm)			L/T
			Min	Max	Means	Min	Max	Means	
No artificial management	1	Top	365.14	2624.38	1181.47 ± 21.36c**	10.55	50.96	23.40 ± 1.30b	50.49
		Middle	869.40	4490.60	2493.17 ± 107.52a**	12.17	50.12	28.07 ± 0.65a**	88.82**
		Bottom	1378.24	3162.28	2125.90 ± 118.00b**	12.83	41.83	21.98 ± 1.15c**	96.73**
		Means	870.93	3425.75	1933.51 ± 82.30**	11.85	47.64	24.48 ± 1.03**	78.98**
	2	Top	799.74	3559.52	1763.08 ± 17.18b	15.25	42.42	25.65 ± 0.79b	68.73
		Middle	719.70	4285.64	1780.92 ± 116.79a**	13.75	61.17	29.73 ± 2.10a	59.90**
		Bottom	708.69	3031.67	1574.24 ± 43.76c**	12.50	55.24	29.94 ± 0.88a	52.58**
		Means	742.71	3625.61	1706.08 ± 59.24	13.83	52.94	28.44 ± 1.26	59.99**
	3	Top	592.93	2588.96	1452.24 ± 38.58b	17.10	39.91	28.36 ± 1.44a	51.22
		Middle	680.21	2889.39	1469.05 ± 17.03ab	15.65	45.09	28.18 ± 0.54a	52.13
		Bottom	466.52	3142.62	1460.92 ± 84.15a	11.33	38.63	23.47 ± 0.33b	62.24
		Means	579.89	2873.66	1460.74 ± 46.59	14.69	41.21	26.67 ± 0.77	54.77
	Means	Top	585.94	2924.29	1465.6 ± 25.71	14.30	44.43	25.80 ± 1.18	56.81
		Middle	756.44	3888.54	1914.38 ± 80.45	13.86	52.13	28.66 ± 3.29	66.95
		Bottom	851.15	3112.19	1720.35 ± 81.97	12.22	45.23	25.13 ± 2.36	70.51
		Means	731.17	3308.34	1700.11 ± 62.51	13.46	47.26	26.53 ± 2.28	64.58
Artificial management	1	Top	679.10	1878.50	1118.49 ± 14.23c	9.84	38.01	22.08 ± 0.81ab	50.66
		Middle	789.54	3073.36	1768.68 ± 256.20a	13.74	40.64	21.50 ± 0.55b	82.27
		Bottom	651.88	2791.22	1509.41 ± 96.59b	8.80	43.73	22.56 ± 0.56a	66.89
		Means	706.84	2581.03	1465.53 ± 122.34	10.79	40.79	22.05 ± 0.64	66.47
	2	Top	–	–	–	–	–	–	–
		Middle	611.18	2270.78	1263.63 ± 49.74a	13.71	43.73	28.89 ± 1.66b	43.74
		Bottom	633.30	1895.22	1187.32 ± 63.43b	13.80	48.25	29.70 ± 1.49a	39.97
		Means	622.24	2083.00	1225.47 ± 56.59	13.76	45.99	29.30 ± 1.57	41.83
	Means	Top	679.10	1878.50	1118.49 ± 14.23	9.84	38.01	22.08 ± 0.81	50.66
		Middle	700.36	2672.07	1516.16 ± 152.97	13.72	42.19	25.20 ± 1.11	63.00
		Bottom	642.59	2343.22	1348.37 ± 80.01	11.30	45.99	26.13 ± 1.03	53.43
		Means	674.02	2297.93	1327.67 ± 82.40	11.62	42.06	24.47 ± 0.98	55.70

1, 2, 3 and Means indicated the values of 1-year-old, 2-year-old and 3-year-old bamboos without and with artificial cultivation and management at different height, and these values of means. * indicated the significant difference (P<0.05) between the bamboos without and with artificial cultivation and management in the same age and internodes. ** indicated extremely significant difference (P<0.01) and even highly significant difference (P<0.001) between the bamboos without and with artificial cultivation and management in the same age and internodes. Lower case letters represented the significance different heights in the same age.

**Table 2:** Changes of the wall thickness, lumen diameter and W/Lu ratio of *D.brandisii* in the two management modes.

Management	Age (year)	Position	Wall thickness (µm)			Lumen diameter (µm)			W/Lu
			Min	Max	Means	Min	Max	Means	
No artificial management	1	Top	1.28	21.58	9.40 ± 0.43b**	0.54	23.60	4.74 ± 0.59c**	3.97**
		Middle	3.82	23.26	11.52 ± 0.30a**	0.36	17.51	5.41 ± 0.63b**	4.26**
		Bottom	2.89	14.93	6.22 ± 0.14c**	3.38	20.70	9.44 ± 0.90a**	1.32**
		Means	2.66	19.92	9.05 ± 0.29**	1.43	20.60	6.53 ± 0.71**	2.77**
	2	Top	3.20	20.31	10.86 ± 0.38c	0.54	19.02	4.45 ± 0.23c	4.88**
		Middle	3.06	24.18	12.52 ± 0.99a**	0.54	23.82	5.12 ± 0.71b**	4.89**
		Bottom	3.46	18.91	11.69 ± 0.41b**	0.54	41.32	6.84 ± 0.64a**	3.42**
		Means	3.24	21.13	11.69 ± 0.59	0.54	28.05	5.47 ± 0.53	4.27**
	3	Top	6.21	17.95	12.52 ± 0.35a	0.36	9.96	3.43 ± 0.68a	7.31
		Middle	5.77	18.75	12.32 ± 0.37ab	0.36	20.20	3.63 ± 0.23a	6.80
		Bottom	3.19	15.30	9.76 ± 0.34b	0.36	21.84	4.23 ± 1.06a	4.61
		Means	5.06	17.33	11.53 ± 0.35	0.36	17.33	3.76 ± 0.66	6.13
	Means	Top	3.56	19.95	10.93 ± 0.39	0.48	17.53	4.21 ± 0.50	5.39
		Middle	4.22	22.06	12.12 ± 0.55	0.42	20.51	4.72 ± 0.52	5.31
		Bottom	3.18	16.38	9.22 ± 0.30	1.43	27.95	6.84 ± 0.87	3.12
		Means	3.65	19.46	10.76 ± 0.41	0.78	22.00	5.26 ± 0.63	4.39
Artificial management	1	Top	2.29	10.45	6.18 ± 0.27a	2.79	21.74	9.74 ± 0.48b	1.27
		Middle	3.12	11.45	5.94 ± 0.09ab	3.12	22.77	9.62 ± 0.62b	1.24
		Bottom	2.64	10.21	5.68 ± 0.20b	2.11	33.98	11.06 ± 0.19a	1.03
		Means	2.68	10.70	5.93 ± 0.19	2.67	26.16	10.14 ± 0.43	1.17
	2	Top	–	–	–	–	–	–	–
		Middle	5.07	17.33	9.77 ± 0.37b	1.52	26.54	9.41 ± 0.85a	2.08
		Bottom	4.74	20.26	10.15 ± 0.58a	1.80	28.25	9.11 ± 0.47a	2.23
		Means	4.90	18.79	9.96 ± 0.48	1.66	27.39	9.26 ± 0.66	2.15
	Means	Top	2.29	10.45	6.18 ± 0.27	2.79	21.74	9.74 ± 0.48	1.27
		Middle	4.10	14.39	7.86 ± 0.23	2.32	24.65	9.52 ± 0.55	1.66
		Bottom	3.69	15.24	7.92 ± 0.39	1.95	31.11	10.09 ± 0.33	1.63
		Means	3.36	13.36	7.32 ± 0.30	2.35	25.83	9.78 ± 0.45	1.52

1, 2, 3 and Means indicated the values of 1-year-old, 2-year-old and 3-year-old bamboos without and with artificial cultivation and management at different height, and these values of means. ** indicated extremely significant difference (P<0.01) and even highly significant difference (P<0.001) between the bamboos without and with artificial cultivation and management in the same age and internodes. Lower case letters represented the significance different heights in the same age.

The study showed that the fibers of the same internode in the bamboos, regardless of whether they were under management or not, gradually became shorter as the age advanced (**Table 1**). This could be attributed to the fact that the height and internode length were greater in 1-year-old culms compared to the 2- and 3-year-old ones. Typically, newly formed bamboo culms exhibited a taller height and larger diameter than their older counterparts. Under the management conditions, the fibers of the culms were significantly shorter than those of the culms without any management, especially in the 1-year-old bamboo culms. According to the means of fiber length, the fibers at the middle parts were the longest among the bamboos of all ages. For 1- and 2-year-old bamboos, the shortest fibers were found at the top, while for 3-year-old bamboos, they were at the bottom. Overall, the order of fiber length was middle> bottom> top.

**Figure 3:**
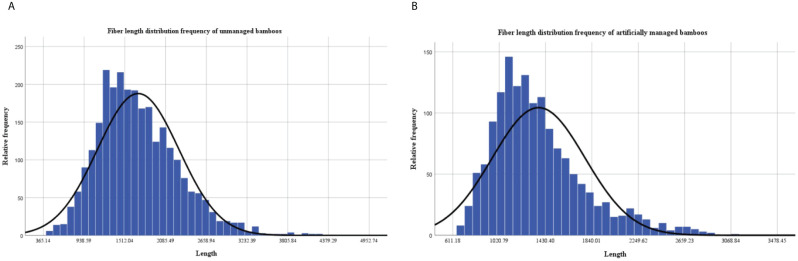
Frequency distribution of fiber length. **(A)** Bamboos without management. **(B)** Bamboos with management.

According to the frequency distribution of fiber lengths, it could be noticed that the fiber length of *D. brandisii* a normal distribution pattern (**Figure 3**). The fibers of the bamboos without management ranged in length from 365.14 to 4490.6 µm, with the majority falling within the range of 938.59 to 2085.49 µm. The average length of these fibers was 1700.11 ± 62.51 µm (**Figure 3A**). While under the management conditions, the fiber length ranged from 611.18 to 3073.36 µm, with the majority falling within the range of 1020.79 to 1430.40 µm, and the average length was 1327.67 ± 82.40 µm (**Figure 3B**).

For the bamboos in the wild field without management, the order of tangential diameter of fibers in 1-year-old culms was middle> top>bottom. For the 2-year-old culms, the order was bottom> middle> top, while the trend in the bamboos of 3 years was completely opposite. This might be due to the differences in the length and diameter of internodes of different bamboos. Generally, the fibers in the middle (28.66 ± 3.29 µm) of the culms had the largest tangential diameter, followed by the top (25.80 ± 1.18 µm) and the bottom (25.13 ± 2.36 µm). However, the fiber tangential diameter of culms under management conditions displayed an opposite trend, with the largest values at the bottom (26.13 ± 1.03 µm), followed by the middle (25.20 ± 1.11 µm) and the smallest values at the top (22.08 ± 0.81 µm). In general, the artificial management significantly decreased the fiber tangential diameters.

For the 1- and 3-year-old culms without management, the order of slenderness ratios (L/T ratio) were bottom> middle> top, while the trend was reversed in 2-year-old bamboos. The highest values appeared at the bottom (70.51), followed by the middle (66.95) and the smallest values at the top (56.81). In addition, the mean values of the L/T ratio gradually decreased with age from 78.98 (1-year-old) to 54.77 (3-year old bamboos). For the bamboos with artificial management, the largest L/T ratios were found at the middle (63.00), followed by the bottom (53.43) and the top (50.66). Generally, the L/T ratio of the bamboos under artificial management conditions showed significant smaller values than those of the bamboos without any management (**Table 1**).

For the fiber wall thickness, the 1- and 2-year-old culms without any management showed larger values in the middle compared to the top and bottom parts (**Table 2**).While in the 3-year-old culms, the fibers in the middle and top showed thicker walls than the bottom. In general, the middle of the culms had the thickest fiber walls (12.12 ± 0.55 µm), followed by the top (10.93 ± 0.39 µm) and the bottom (9.22 ± 0.30 µm). Additionally, the fiber wall thickness firstly increased and then slightly decreased with age with the highest values in the culms of 2 years. For the bamboos with artificial management, the fiber wall thickness also showed higher values in 2-year-old culms than in 1-year-od culms. Additionally, the fibers in the culms showed thicker walls at the bottom (7.92 ± 0.39 µm) and middle (7.86 ± 0.23 µm) than the top (6.18 ± 0.27 µm) parts. Accordingly, the bamboos with management showed thinner fiber wall thickness than those bamboos lacking management.

The fiber lumen diameter showed a decreasing trend with age in the culms with or without management (**Table 2**). Additionally, the decreasing trends were also shown along the culms from the bottom to the top. In addition, it was observed that the culms with management showed significant larger lumen diameters compared to those without any management.

As for the Runkel ratio (W/Lu), the values were higher in the top and middle than the bottom in the culms of all ages (**Table 2**). Meanwhile, the ratios also increased constantly with age with the highest values in the 3-year-old culms, which also revealed the constant fiber wall deposition and the constant decrease of lumen diameter with age.

### Moisture content and chemical composition of *D. brandisii* culms with and without artificial management

**Figure 4:**
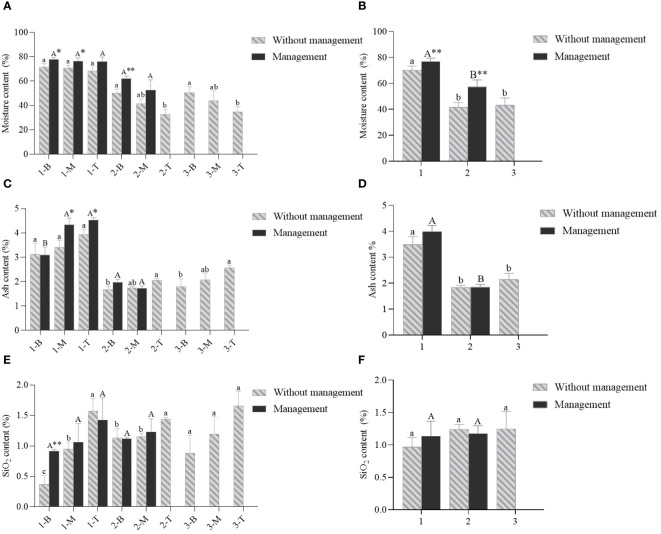
The contents of moisture, ash and SiO_2_ of *D. brandisi* culms without and with management. **(A)** Moisture content of different portions of culms under two management modes. **(B)** Moisture content of different ages of culms under two management modes. **(C)** Ash content of different portions of culms under two management modes. **(D)** Ash content of different ages of culms under two management modes. **(E)** SiO_2_ content of different portions of culms under two management modes. **(F)** SiO_2_ contents of different ages of culms under two management modes. 1 indicated 1-year-old bamboos. 2 indicated 2-year-old bamboos. 3 indicated 3-year-old bamboos. B, M and T indicated the bottom, middle and top of bamboos, respectively. Different lowercase letters indicated significant differences (P<0.05) in the unmanaged bamboos at the same age. Different uppercase letters indicated significant differences (P<0.05) in the artificially managed bamboos at the same age. * indicated significant difference (0.01<P<0.05) between two management modes. ** indicated extremely significant difference (P<0.01) between two management modes.

Moisture played an important role in the growth and development of bamboos. The moisture content of *D. brandisii* culms showed a constantly decreasing trend from the bottom to the top in the culms of all ages, but the differences were not significant (**Figure 4A**). Additionally, the moisture content in the culms lacking management decreased significantly from 1-year to 2-year-old culms and then slightly increased in the 3-year-old culms (**Figures 4A, B**). Under management conditions, the moisture contents also decreased in the culms from 1 year to 2 years. Additionally, the moisture content was significantly higher in the bamboos with management than in those lacking management.

**Figure 5:**
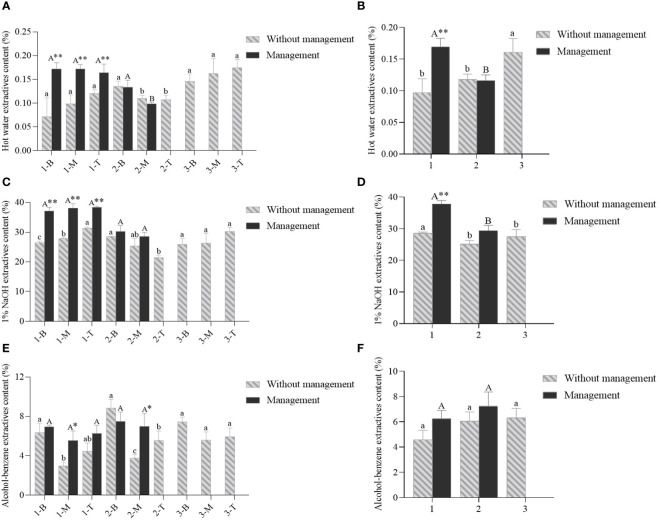
The contents of hot water, 1% NaOH and alcohol-benzene extractives of *D. brandisi* culms without and with management. **(A)** The hot water extractives content of different portions of culms under two management modes. **(B)** The hot water extractives content of different ages of culms under two management modes. **(C)** The 1% NaOH extractives contents of different portions of culms under two management modes. **(D)** The 1% NaOH extractives contents of different ages of culms under two management modes. **(E)** The alcohol-benzene extractives contents of different portions of culms under two management modes. **(F)** The alcohol-benzene extractive contents of different ages of culms under two management modes. 1 indicated 1-year-old bamboos. 2 indicated 2-year-old bamboos. 3 indicated 3-year-old bamboos. B, M and T indicated the bottom, middle and top of bamboos, respectively. Different lowercase letters indicated significant differences (P<0.05) in unmanaged bamboos at the same age. Different uppercase letters indicated significant differences (P<0.05) in artificially managed bamboos at the same age. * indicated significant difference (0.01<P<0.05) between two management modes. ** indicated extremely significant difference (P<0.01) between two management modes.

**Figure 6:**
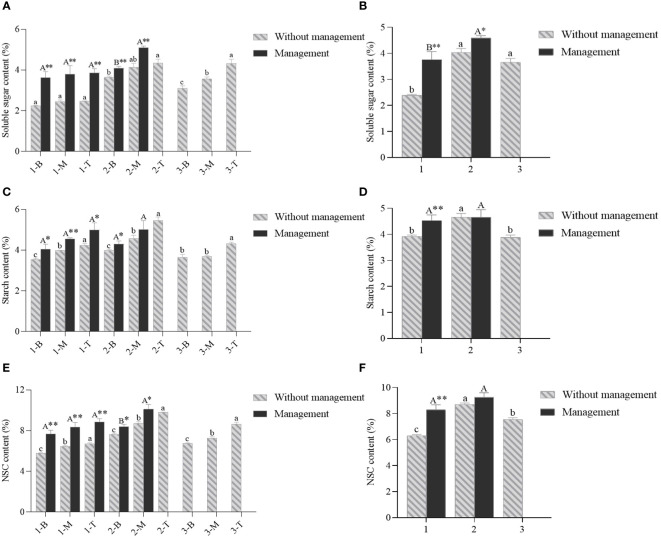
The contents of holocellulose, acid-soluble lignin and acid-insoluble lignin of *D. brandisi* culms without and with management. **(A)** The holocellulose content of different portions of culms under two management modes. **(B)** The holocellulose content of different ages of culms under two management modes. **(C)** The acid-soluble lignin content of different portions of culms under two management modes. **(D)** The acid-soluble lignin content of different ages of culms under two management modes. **(E)** The acid-insoluble lignin content of different portions of culms under two management modes. **(F)** The acid-insoluble lignin contents of different ages of culms under two management modes. 1 indicated 1-year-old bamboos. 2 indicated 2-year-old bamboos. 3 indicated 3-year-old bamboos. B, M and T indicated the bottom, middle and top of bamboos, respectively. Different lowercase letters indicated significant differences (P<0.05) in unmanaged bamboos at the same age. Different uppercase letters indicated significant differences (P<0.05) in artificially managed bamboos at the same age. * indicated significant difference (0.01<P<0.05) between two management modes. ** indicated extremely significant difference (P<0.01) between two management modes.

The chemical composition of culms was measured and compared between the bamboos with management and those lacking management (**Figures 4**–**6**). The ash contents showed a constantly increasing trend from the bottom to the top in the culms of all ages, but the differences were not significant (**Figure 4C**). Meanwhile, the ash contents also showed apparent trend in the bamboo culms with age, which first decreased significantly and then increased with age in the culms lacking management (**Figures 4C, D**). The ash content trend was similar in the culms with management, with an increase from 1 year to 2 years old. Generally, the culms under management conditions showed higher ash contents in all internodes, particularly in 1-year-old culms. This indicated that the artificial management could increase the ash contents of bamboos. Similar to the ash contents, the SiO_2_ contents also showed a constantly and slightly increasing trend from the bottom to the top in the culms of all ages (**Figures 4E, F**). In addition, the management increased the SiO_2_ content in 1-year-old culms, but reduced that in the 2-year-old culms.

It was shown in the bamboos without management that the contents of hot water extractives gradually increased from the bottom to the top in the 1- and 3-year-old bamboos (**Figure 5A**). However, in the 2-year-old bamboos, the opposite trend was observed with a gradual decrease in hot water extractives contents. In addition, the contents of hot water extractives showed a constantly increasing trend with age (**Figures 5A, B**). However, the artificial management significantly increased the hot water extractives contents in 1-year-old culms, but had no significant influences on the content in 2-year-old culms. The same pattern was also observed in the contents of 1% NaOH extractives that constantly increased from the bottom to the top in the culms of all ages, except in the 2-year-old culms, which constantly decreased along the culms (**Figures 5C, D**). Unlike the hot water extractives, the contents of 1% NaOH extractives showed lower values in 1-year-old culms as compared to those in 1- and 3 year-old culms. Additionally, the bamboos with artificial management showed higher contents of 1% NaOH extractives than those bamboos lacking management, which revealed that the artificial management had a significant impact on the 1% NaOH extractives contents in bamboos.

As for the content of alcohol-benzene extractives, it was the highest at the bottom, followed by the top and the lowest in the middle in the bamboos without managements as well as those with managements (**Figure 5E**). It was also shown that the content of alcohol-benzene extractives constantly increased with age in both the bamboos with and without managements (**Figure 5F**). Meanwhile, the bamboo culms with managements also showed higher contents as compared to those without managements. Generally, it concluded that the artificial management could significantly increase the contents of all kinds of extractives.

The holocellulose contents constantly and slightly decreased with height in the bamboos of all ages with the highest values at the bottom in the bamboo culms without managements, but no apparent trends were observed in the bamboo culms with managements (**Figure 6A**). It could also be observed that holocellulose contents slightly and constantly increased with age, and the contents were always higher in the bamboos with managements than those without managements (**Figure 6B**).

The bamboo culms lacking managements showed the highest values of acid-insoluble lignin contents in the middle parts, followed by the top and the middle parts in the bamboo culms of all ages (**Figure 6C**). The acid-insoluble lignin contents also showed an increasing trend with age in the bamboo culms without managements. A similar trend was also shown in the culms with managements (**Figure 6D**). However, the acid-insoluble lignin contents were always lower in the culms with management than in those without managements (**Figure 6D**).

As for acid-soluble lignin, the contents showed an increasing trend with height in the culms of all ages with the highest values in the top (**Figure 6E**). It was also observed that the contents of acid-soluble lignin constantly increased with age, and the contents of which was also lower in the bamboo culms with managements than in those lacking managements (**Figure 6F**). Generally, the artificial management decreased the contents of acid-insoluble and acid-soluble lignins, but increased the holocellulose contents in bamboos.

### Soluble sugar, starch and NSC contents of *D. brandisii* culms with and without management

**Figure 7:**
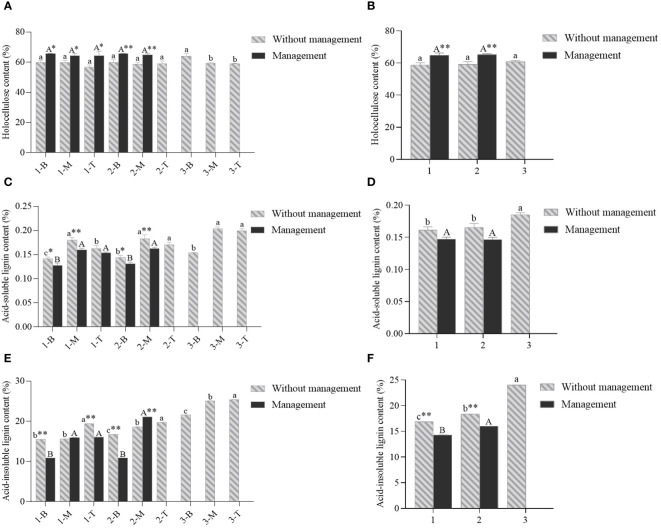
The contents of soluble sugar, starch and NSC of *D. brandisi* culms without and with management. **(A)** The content of soluble sugar of different portions of culms under two management modes. **(B)** The soluble sugar content of different ages of culms under two management modes. **(C)** The starch content of different portions of culms under two management modes. **(D)** The starch content of different age of culms under two management modes. **(E)** The content of NSC of different portions of culms under two management modes. **(F)** The NSC contents of different age of culms under two management modes. 1 indicated 1-year-old bamboos. 2 indicated 2-year-old bamboos. 3 indicated 3-year-old bamboos. B, M and T indicated the bottom, middle and top of bamboos, respectively. Different lowercase letters indicated significant differences (P<0.05) in unmanaged bamboos at the same age. Different uppercase letters indicated significant differences (P<0.05) in artificially managed bamboos at the same age. * indicated significant difference (0.01<P<0.05) between two management modes. ** indicated extremely significant difference (P<0.01) between two management modes.

The contents of soluble sugar, starch and NSC in the *D. brandisii* culms under two management conditions were measured and compared to analyze the influences of artificial management on sugar accumulation in bamboo culms (**Figure 7**). It could be noticed that in the three ages of bamboos, the contents of soluble sugar and starch gradually increased from the bottom to the top (**Figures 7A–D**). In addition, the variations in soluble sugar and starch contents were consistent in the bamboo culms lacking managements, which increased significantly at first and then decreased as the bamboo matured and aged (**Figures 7A, B**). The same trend appeared in the culms with management. Furthermore, the soluble sugar and starch contents of artificially managed culms were significant higher than those without management, especially in the 1-year-old culms (**Figures 7A–D**). The NSC content followed the same trend, with the highest values in 2-year-old culms. Generally, the artificial management increased the carbohydrates storage in the culms (**Figures 7E, F**).

## Discussion

The practice of utilizing bamboo instead of wood as the primary material for paper production has become exceedingly prevalent. A variety of bamboo species such as *Fargesia edulis, D. hamiltonii*, *Ph. edulis*, *D. membranceus*, *B. textilis* and *D. yunnaicus* had been reported to be suitable for paper-making (Feng et al., 2005; Yang et al., 2007, 2008; Tang et al., 2015; Zhan et al., 2017b; Li et al., 2019; Xiang et al., 2019). *D. brandisii* was one of the most popular species to cultivate for edible shoots. The selective thinning and top trimming were extensively used during the management of *D. brandisii* stand. However, only a limited number of studies have been conducted on the effects of these management practices on bamboos, resulting in a lack of sufficient theoretical support for the benefits of such management techniques in bamboo forest management.

### Differences in fiber morphology between *D. brandisii* culms with management and without management

Fiber morphology was one of the fundamental characteristics of paper-making plant raw materials, which encompassed factors such as fiber length, tangential diameter, wall thickness, lumen diameter, slenderness ratio and Runkel ratio. These indicators not only served as a crucial foundation for analyzing the advantages and disadvantages of paper-making raw materials, but also for determining the process conditions. Furthermore, they were significant factors that influenced the quality of bamboo pulp paper strength, as reported by Somwang et al. (2022).

The frequency of fiber length distribution was a crucial indicator for evaluating the quality of paper-making raw materials (Masubuchi et al., 2016). According to the fiber length, the fibers were usually categorized into four groups, among which fibers shorter than 0.90 mm belonged to short fibers, and fibers between 0.90 and 1.60 mm were considered as medium-long fibers, as well as fibers longer than 1.60 mm belonged to long fibers, and fibers with a length greater than 3.00 mm were considered as extremely long fibers (Yang, 2005). In general, the pulp quality was superior when the fibers were longer. The fibers of *D. brandisii* culms without management reached 1700 ± 62.51µm, which belonged to the long fibers. This length was shorter than the fibers of other bamboo species, such as *F. edulis* (1.79 mm), *B. balcooa* (2.18 mm), *B. arundinacea* (2.01 mm), *Dendrocalamopsis oldhami* (2.19 mm), and *Ph. bambusoides* (2.30 mm), but longer than those of *Populus deltoides* (1.11 mm) (Cao et al., 2014; Tang et al., 2015; Azeez et al., 2016; Deniz et al., 2017; Liu et al., 2020). Under management conditions, the fiber length was 1.33 mm, which was significantly lower than that of the culms without management, but still longer than those of *P. deltoides* and belonged to the medium fibers. Therefore, the artificial management decreased the fiber length.

The tangential diameter of fibers was intimately linked to the cross-sectional area, and specifically, fibers with a wider cross-section possessed a larger cross-sectional area, ultimately enhancing the production of high-quality and high-strength paper (Anupam et al., 2016). The tangential diameter of *D. brandisii* fibers (26.53 ± 2.28µm) was significantly larger than those of *B. emeiensis* (15.00 µm), *Ph. edulis* (16.20 µm), *F. edulis* (14.25 µm) (Yang et al., 2008; Tang et al., 2015). Generally, the artificially managed bamboos were thinner and shorter than unmanaged bamboos, so we speculated that the diameters of fibers were limited by the length and diameter of bamboo internodes. Usually, the higher the L/T ratio of the fiber, the higher the paper strength (Masubuchi et al., 2016). Pulp fiber L/T ratio should be > 45, and a higher ratio was preferred during papermaking process (Wang et al., 2008; Yang et al., 2008). In the wild field, the mean L/T ratio of *D. brandisii* fibers was 64.58, whereas after artificial management, it decreased to 55.70, which still fulfills the requirements for paper-making raw material.

The W/Lu ratio of fiber was used to indicate the softness of the fiber (Masubuchi et al., 2016). The low W/Lu ratio indicated that the fibers with large cavity diameter and thin wall had excellent softness and exceptional paper strength (Casey, 2009). This study noticed that the W/Lu ratio of the fibers in *D. brandisii* culms without management was 4.39, which was slightly lower than that of the fibers of *B. textilis* (5.90), but higher than that of the fibers of *B. sinospinosa* (3.77), *D. minor* (3.11) and *B. pervariabilis* (4.00) (Su et al., 2011; Xiang et al., 2019). After the artificial management, the W/Lu ratio values decreased to be 1.52, which could enhance the paper strength.

The artificial management decreased the length, tangential diameter and L/T ratio of fibers, but also decreased the wall thickness and increased the lumen diameter and W/Lu ratio of the fibers. Therefore, the *D. brandisii* culms could still be used as paper-making raw materials after managements.

### Differences in chemical composition between *D. brandisii* culms with management and without management

With the maturation of young bamboos, the moisture content decreased constantly and significantly (Liu et al., 2014). Once the culms completed their height growth, the moisture content stabilized and no longer fluctuated greatly (Liu et al., 2023). Zhan et al. (2015) reached the same conclusion. Zhan et al. (2015) considered that the decrease in moisture content with age might be connected to the lignification of vascular bundles and parenchyma cells. The anatomical structure of *D. brandisii* also verified this statement, in which the lignification degree of fiber and parenchyma cells increased constantly according to the microscopic observations.

The ash content in wood was generally lower than 2.0% (Pazalja et al., 2021). A high ash content could cause problems such as refining and in the recovery system during the pulping process (Liu et al., 2014; Gülsoy and Şimşir, 2018). The SiO_2_ content constantly increased with age in the form of phytolith in bamboos (Liu et al., 2023). Generally, as the age increased, the accumulation of various minerals in the surface layer of bamboo wall led to higher contents of ash and SiO_2_ in the surface layer of bamboo wall (Liu et al., 2023). The increase of ash and SiO_2_ contents in the *D. brandisii* bamboos with artificially managements might be due to the fact that the selective thinning and trimming might enhance the ability in inorganic salt absorption, which further led to an increase in ash and SiO_2_ contents in the culms. The processing and soda recovery during processing would be adversely affected by the higher ash and SiO_2_ contents in the bamboos.

The low contents of 1% NaOH extractives and alcohol-benzene extractives material made it easier for chemicals to penetrate the material and could be usually used to produce high-quality paper (Fatriasari and Hermiati, 2008). High contents of 1% NaOH extractives and alcohol-benzene extractives increased the use of chemicals in pulping process (Liu et al., 2014). 1% NaOH extract could not only dissolve the substances dissolved in hot water, such as sugar, starch, amino acid, inorganic salt and tannin, but also dissolved some lignin, pentosan and resin acid (Liu et al., 2014). The 1% NaOH extractives content of *D. brandisii* was significantly higher than that of *D. hamiltonii* (17.24%), *B. emeiensis* (24.27%), *B.textilis* (28.38%), *B. vulgaris* (24.44%) and *D. membranaceus* (23.20%), and was similar to that of *B. sinospinosa* (33.33%) and *D. yunnanicus* (40.05%) (Yang et al., 2007; Su et al., 2011; Somwang et al., 2022). With the growth of *D. brandisii*, the content of 1% NaOH extractives first decreased and then increased, which was inconsistent with that reported by Zhan et al. (2015) and Liu et al. (2023).

The components of alcohol-benzene extractives mainly included pigments, fatty acids, resin acids, waxes and phenolic compounds (Santos et al., 2022). These substances were often referred to as “resins” in the pulp industry (Yang et al., 2007). The *D. brandisii* had higher alcohol-benzene extractives content compared to other bamboo species, including *D. hamiltonii* (2.65%), *B. sinospinosa* (2.47%), and *B. emeiensis* (1.24%), and was similar to that of *D. yunnanicus* (5.22%) and *B. textilis* (5.81%) (Su et al., 2011; Somwang et al., 2022). However, the artificial management further increased the extractives contents in the bamboo culms, such as sugar, starch, and inorganic salts, which might decreased the pulp yields. This might be due to the fact that the selective thinning and top trimming enhanced the capacities in nutrient synthesis and absorption.

High-quality paper was usually produced from materials with high comprehensive cellulose content, while materials with high lignin content could consume large amounts of chemical reagents in the pulping process (Afrifah et al., 2022). The contents of holocellulose and lignin increased with age. The gradual secondary wall deposition and lignification of fiber cells and parenchyma cells, coupled with the high growth of bamboo, mainly accounted for this occurrence. Thus the contents of holocellulose and lignin gradually increased with age (Gritsch et al., 2004; Wang et al., 2011, 2016). The wood used for papermaking typically consisted of coniferous woods and broad-leaved woods, with holocellulose contents usually 65% -73% and 70% -82% (Su et al., 2011). As compared to the bamboos without managements, the managed *D. brandisii* bamboos showed higher holocellulose contents but lower acid-soluble and insoluble lignin contents. Therefore, the managed *D. brandisii* bamboos were still suitable for paper making. However, because of the increased contents of ash contents and extractives, utilizing the managed *D. brandisii* culms for paper making might lead to increased alkali consumption and difficulty to recover.

### Differences in carbohydrates storage between *D. brandisii* culms with management and without management

The main forms of carbohydrates stored in plant vegetative tissues were soluble sugar and starch, which were also the main nutrients consumed in bamboo (Khalil et al., 2006). Typically, 1-year-old bamboos served as the mother bamboo for the subsequent year’s shooting, supplying ample NSC and water for the new germinated shoot growth (Li et al., 2023b). The 2-year-old bamboos were part of the adult bamboos with lush branches and leaves, and the main function of which was to carry out photosynthesis, thereby supplying nutrients for the entire bamboo clusters. Conversely, the 3-year-old or older bamboos could compete more nutrients than supply compared with the younger bamboos (Shi et al., 2023). Hui and Yang (2002) presented that the proper artificial selective cutting and fertilization could increase the photosynthetic area reasonably, which facilitated the accumulation of soluble sugar and starch in bamboos. Therefore, the contents of soluble sugar, starch and NSC in the artificially managed *D. brandisii* bamboos were significantly higher than those lacking management. The anatomical observations also supported this result that the number of starch grains significantly increased in the bamboos after the artificial management. Therefore, the artificial cultivation and management could effectively enhance the NSCs storage in the *D. brandisii* culms, and then further increased the shoot production of the next year. However, Okahisa et al. (2006) considered that the perishable properties of bamboo are mainly caused by its high sugar and starch contents, which are foods for fungi or insects. Therefore, the increased NSCs contents in culms might improved the difficulty in storage.

Generally, under artificial management conditions, the bamboo culms of *D. brandisii* remain suitable for timber applications. However, it is also worthy of mention that utilizing these manually managed bamboo culms for timber, particularly in the production of paper, might pose a potential risk of mold growth or insect infestation in the final products. Furthermore, the observed elevation in NSC content within managed bamboo culms suggested that effective management practices could facilitate sugar accumulation in these culms, ultimately fostering the germination of bamboo shoots in the subsequent year. It was noteworthy that the study lacked a comprehensive investigation into the impact of artificial management techniques on pest and disease control, their influence on ground biomass, and their role in shaping soil microbial communities in D*. brandisii*. Future research endeavors should delve deeper into these facets of artificial management techniques to gain a more comprehensive understanding.

## Conclusion

Artificial management had a great impact on the fiber morphology and chemical composition of *Dendrocalamus brandisii*, especially on the 1-year-old bamboos. Despite reductions in fiber length, tangential diameter, and L/T ratio, the artificial management successfully led to an increase in lumen diameter, a decrease in wall thickness, and a reduction in the W/Lu ratio of the fiber. Additionally, the artificial management not only increased the contents of ash and SiO_2_, and but also increased the contents of all kinds of extractives. However, the artificial management also reduced the lignification of bamboos and decreased the content of lignin, but increased the content of cellulose. The bamboo culms with artificial management could still be utilized as a raw material for paper production because of the increase of cellulose contents and the decrease of lignin contents. The artificial management could significantly improve the NSCs storage in bamboo culms, thereby further increasing the shoot production of the next year. However, the high NSCs content might increase the difficulty of storing paper.

## Data availability statement

The original contributions presented in the study are included in the article/supplementary material. Further inquiries can be directed to the corresponding author.

## Author contributions

YY: Data curation, Investigation, Resources, Validation, Writing – original draft, Writing – review & editing. CY: Resources, Writing – original draft. YW: Investigation, Methodology, Resources, Software, Supervision, Writing – review & editing. JL: Methodology, Software, Supervision, Writing – review & editing. SW: Funding acquisition, Investigation, Methodology, Resources, Supervision, Writing – review & editing.
